# Ionic Species Affect the Self-Propulsion of Urease-Powered Micromotors

**DOI:** 10.34133/2020/2424972

**Published:** 2020-07-27

**Authors:** Xavier Arqué, Xavier Andrés, Rafael Mestre, Bernard Ciraulo, Jaime Ortega Arroyo, Romain Quidant, Tania Patiño, Samuel Sánchez

**Affiliations:** ^1^Institute for Bioengineering of Catalonia (IBEC), The Barcelona Institute of Science and Technology (BIST), Baldiri i Reixac 10-12, 08028 Barcelona, Spain; ^2^Institute of Photonic Sciences (ICFO), The Barcelona Institute of Science and Technology, Carl Friedrich Gauss 3, 08860 Castelldefels, Barcelona, Spain; ^3^Institució Catalana de Recerca i Estudis Avançats (ICREA), Pg. Lluís Companys 23, 08010 Barcelona, Spain; ^4^Chemistry Department, University of Rome, Tor Vergata, Via della Ricerca Scientifica, 00133 Rome, Italy

## Abstract

Enzyme-powered motors self-propel through the catalysis of *in situ* bioavailable fuels, which makes them excellent candidates for biomedical applications. However, fundamental issues like their motion in biological fluids and the understanding of the propulsion mechanism are critical aspects to be tackled before a future application in biomedicine. Herein, we investigated the physicochemical effects of ionic species on the self-propulsion of urease-powered micromotors. Results showed that the presence of PBS, NaOH, NaCl, and HEPES reduced self-propulsion of urease-powered micromotors pointing towards ion-dependent mechanisms of motion. We studied the 3D motion of urease micromotors using digital holographic microscopy to rule out any motor-surface interaction as the cause of motion decay when salts are present in the media. In order to protect and minimize the negative effect of ionic species on micromotors' performance, we coated the motors with methoxypolyethylene glycol amine (mPEG) showing higher speed compared to noncoated motors at intermediate ionic concentrations. These results provide new insights into the mechanism of urease-powered micromotors, study the effect of ionic media, and contribute with potential solutions to mitigate the reduction of mobility of enzyme-powered micromotors.

## 1. Introduction

The use of enzymes as catalytic engines for the propulsion of micro- and nanomotors offers unique opportunities in biomedicine thanks to their biocompatibility, versatility, and fuel bioavailability [1–3]. Recently, several milestones in the development of enzymatic micro- and nanomotors have been achieved, including enhanced drug delivery [4–8], tissue penetration [9–12], tumor targeting [13], chemotaxis [14–17], and sensing [18–20] capabilities. The rapid advances in the field towards applications are currently demanding for a deeper understanding of the fundamental aspects underlying enzyme-driven propulsion to ensure an efficient design and implementation. In this regard, recent studies have reported that the size [21, 22], particle architecture [23, 24], enzyme distribution [25], and enzyme properties [26–30] are key features that modulate the self-propulsion of micro- and nanomotors. However, while most of studies have focused on the micromotor design, the effect of the surrounding microenvironment has received less attention. One of the main components of biological media are ionic species, which have shown to significantly reduce the speed of platinum-based micromotors, a well-established and characterized model of catalytic micromotors [31–37]. These observations opened a considerable scientific debate on the propulsion mechanism underlying the motion of Pt-based micromotors.

Although neutral self-diffusiophoresis was initially proposed as the mechanism of catalytic swimmers, where the asymmetric gradient of products upon catalysis leads to self-propulsion [38–40], this mechanism could not explain how ions reduced the speed of Pt-based micromotors. Other mechanisms sensitive to electrolyte species could explain this ionic effect, such as ionic self-diffusiophoresis, in which the gradients are generated by ionic products, or the self-electrokinetic mechanism, where motion relies on electron flows between regions of a particle due to reduction and oxidation reactions [31–34].

Interestingly, the effect of electrolytes on self-propulsion was mitigated for a self-electrokinetic micromotor through a polyelectrolyte coating [41]. In this study, the change in surface charge was applied to increase the conductivity of silicon nanowire microswimmers to facilitate the electron flow and increase ion tolerance. This work demonstrated that applying a coating to catalytic nanomotors is a useful strategy to improve their motion in ionic solutions, paving the way to designing nanomotors which can tolerate ionic media in future biomedical applications.

An intriguing and yet unresolved question is whether biocompatible enzymatic micromotors respond similarly to ionic media. It is worth mentioning that different motion dynamics can be observed depending on the particle size [21, 42]. Whereas submicron particles display enhanced diffusion, micron-sized motors show a propulsive and directional motion [21, 43], like Pt-based Janus micromotors.

Nanosized enzymatic motors show enhanced diffusion in different ionic media, including phosphate buffer [16, 30] and phosphate-buffered saline (PBS) [11]. Envisioning the possibility of future biomedical applications against bladder cancer, enzymatic nanomotors have also been reported to actively diffuse in simulated and real urine [13]. In a fundamental research, Somasundar and coworkers reported the effect of ionic species on the direction of motion of enzyme-coated liposomes, observing chemotactic behavior based on enzymatic activity and the interaction between ionic species and the nanomotor surface [15].

Although nanosized motors do show enhanced diffusion in ionic media, micron-sized motors present directional motion for longer time scales, and hence, the effect of electrolytes could be rather different [21, 42, 43]. Thus, determining the effect of ionic species on the motion of micron-sized enzymatic motors is of relevance in the field, not only to evaluate their performance in ionic media but also to compare with previous fundamental studies and elucidate their motion mechanism [21, 31, 32, 35, 38]. Herein, we investigate the speed of urease-powered micromotors in physiological ionic conditions (PBS), pH-basic (NaOH), and pH-neutral (NaCl) individual salts and an acidic pH buffer (HEPES). To ensure that there is no physical effect of the bottom surface on motion, we check propulsion through 3D digital holographic microscopy. Given the limitations that ionic media represent for ion-dependent micromotors, we propose a polymeric coating modification for improving their motion performance.

## 2. Results

### 2.1. Fabrication and Characterization of Urease Micromotors

**Figure 1:**
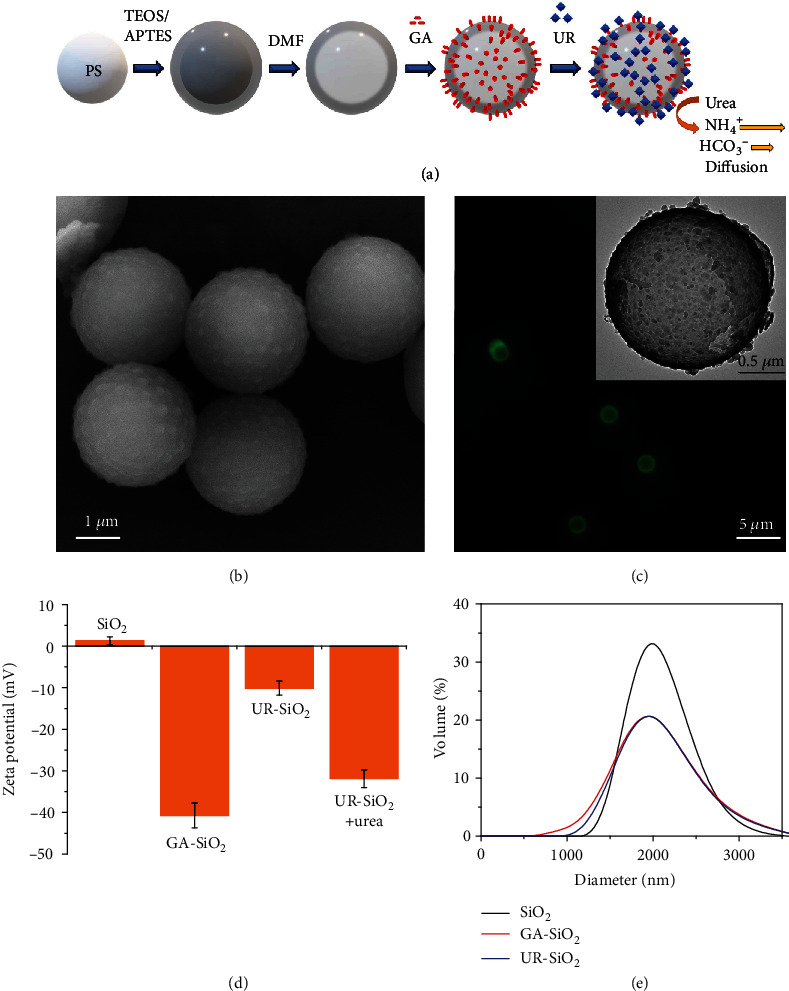
Fabrication and characterization of urease hollow silica micromotors. (a) Schematic of the fabrication process of urease micromotors. (b) SEM of the surface roughness of the hollow silica microcapsules. (c) Fluorescent (Ex/Em = 520/580 nm) Krypton™ protein staining of the urease attached to the microcapsule surface. Inset: TEM micrograph of a microcapsule. (d) Zeta potential of the microcapsules on each step of the fabrication process and after adding urea (substrate). Results are shown as the mean ± standard error of the mean. (e) Hydrodynamic diameter measurements of the microcapsules on each step of the fabrication process.

Micromotors were fabricated as previously reported (see Supplementary Materials) [26]. Briefly, a silicon dioxide (SiO_2_) shell was grown onto commercial 2 *μ*m particles based on polystyrene (PS), using tetraethylorthosilicate (TEOS) and 3-aminopropyltriethoxysilane (APTES) as silica precursors. The PS core was then removed using N,N-dimethylformamide (DMF) to obtain hollow silica microcapsules (Figure 1(a)). Morphology of the resulting capsules was characterized by using scanning and transmission electron microscopy (SEM and TEM, respectively), revealing a highly rough surface (Figures 1(b) and 1(c), inset). To provide the microcapsules with self-propulsion, we attached urease (UR) to their surface by using glutaraldehyde (GA) as a linker. We confirmed the presence of enzyme onto the microcapsule surface by Krypton™ fluorescent protein staining (Figure 1(c) and Figure S1) and by total protein quantification of unattached enzyme (Figure S2). We monitored the changes in surface charge after each step of the functionalization process by measuring the electrophoretic mobility of the particles (Figure 1(d)). The zeta potential of the synthesized microcapsules was positive due to the presence of amino groups on the silica surface, which shifted to −41 ± 3 mV after their modification with GA and to −10.1 ± 1.7 mV after urease attachment. Upon addition of fuel (200 mM urea), the zeta potential decreased to −33.1 ± 2.1 mV and was maintained for 5 days (Figure S3). We measured the hydrodynamic diameter of the particles by dynamic light scattering (DLS), obtaining a mean of 2.00 ± 0.02 *μ*m (Figure 1(e) and Figure S4). For all motion experiments, urease micromotors were recorded at 25 FPS for 30-35 seconds and at tens of microns above the bottom surface; hence, no physical interaction with any wall was interfering with the self-propulsion in the bulk (Movies 1-4). The videos were analyzed using a custom-designed Python-based software, to extract the trajectories of micromotors, the mean squared displacement (MSD), and the speed (see Supplementary Materials).

### 2.2. Urease Micromotors in Phosphate-Buffered Saline

**Figure 2:**
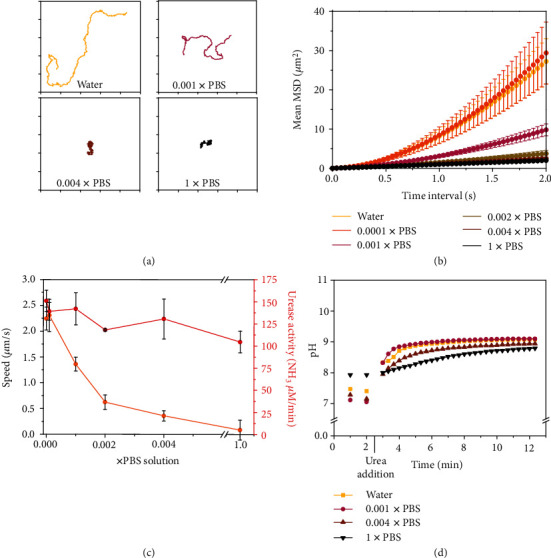
Motion dynamics, enzymatic activity, and pH change produced by urease micromotors for different concentrations of phosphate-buffered saline (PBS) at 200 mM urea. (a) Representative trajectories (axis divided in 10 *μ*m). (b) Average micromotor MSDs. (c) Average speed and enzymatic activity of micromotors. In (b, c), results are shown as the mean ± standard error of the mean. (d) pH change produced by micromotors.

To simulate the ionic strength of biological fluids, we used different concentrations of phosphate-buffered saline (PBS), which consists of an isotonic solution containing physiologically relevant ionic species (NaCl, KCl, Na_2_HPO_4_, and KH_2_PO_4_). We observed a significant decrease in the micromotor's motion when increasing PBS concentration (Figures 2(a)–2(c) and Movie 5). The speed decreased to 0.03 ± 0.19 *μ*m s^−1^ at 1x PBS (137 mM NaCl, 2.7 mM KCl, 10 mM Na_2_HPO_4_, and 1.8 mM KH_2_PO_4_), where the osmolarity and ion concentrations match those of human body fluids. We also determined urease activity through a commercially available colorimetric assay kit, based on the Berthelot method [44], to understand whether the decrease in speed was caused by an inhibition of enzymes by PBS. A slight decrease in urease activity was detected comparing the reaction in water and 1x PBS (Figure 2(c), red). Recently, several studies reported an immediate pH change in the surrounding microenvironment of micro- and nanomotors upon urease activation, due to the ammonia release [10, 18]. To investigate whether the motion reduction was an effect of pH, we monitored pH changes during the reaction. Figure 2(d) shows that upon the addition of urea, the pH shifted quickly (1 min) from 7.4 to around 9 for all conditions. The higher the PBS concentration, the slower the increase of pH (Figure 2(d)).

### 2.3. Urease Micromotors in NaOH

**Figure 3:**
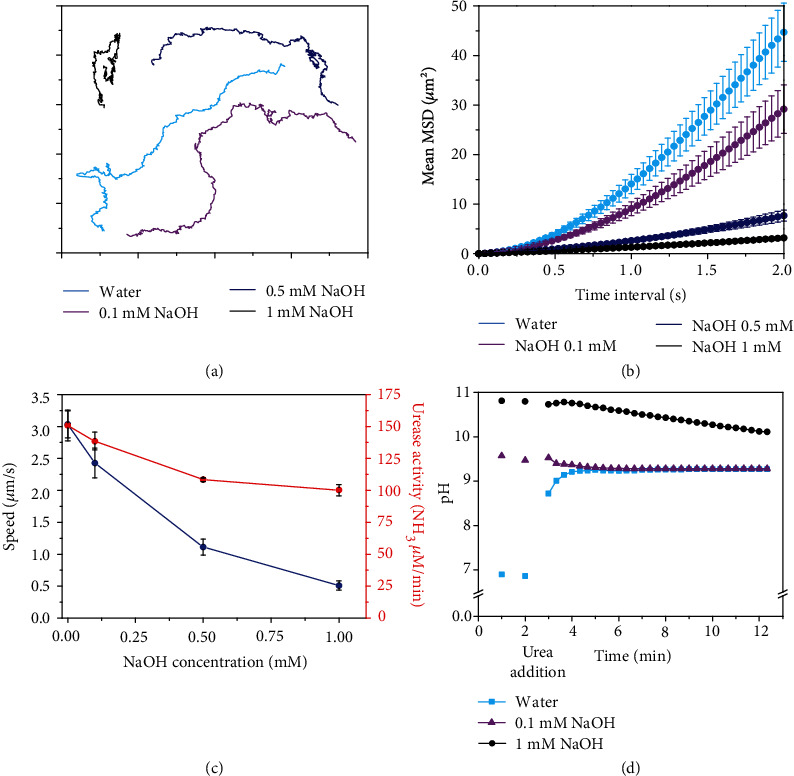
Motion dynamics, enzymatic activity, and pH change produced by urease micromotors for different concentrations of NaOH at 200 mM urea. (a) Representative trajectories (axis divided in 5 *μ*m). (b) Average micromotor MSDs. (c) Average speed and enzymatic activity of micromotors. In (b, c), results are shown as the mean ± standard error of the mean. (d) pH change produced by micromotors.

Since Na^+^ is the main ion present in PBS, we further investigated the effect of a basic (NaOH) salt on the motion of urease micromotors. Increasing NaOH concentrations provoked a decreasing trend on speed, in agreement with the results obtained in PBS (Figures 3(a)–3(c) and Movie 6). The speed decreased from 3.0 ± 0.2 *μ*m s^−1^ in water to 0.51 ± 0.07 *μ*m s^−1^ at 1 mM NaOH (Figure 3(c)), representing a reduction of 83.23%. In these conditions, the enzymatic activity decreased (Figure 3(c), red) by only 33.6%. Moreover, the presence of NaOH increased the pH, reaching a maximum value of 11 at 1 mM NaOH (Figure 3(d)). Thus, the observed reduction of enzyme activity could be attributed to a suboptimal pH for enzyme kinetics [18].

### 2.4. Urease Micromotors in HEPES

Since pH 6 is the optimal pH for the catalytic activity of urease micromotors [18], we also tested the motion of micromotors for different concentrations of an acidic and ionic buffer, namely, HEPES (Figure S5). The initial pH measured for 1 mM HEPES was the most acidic (pH = 6), and it increased slower than in any other experiments upon addition of urea (Figure S5d). Enzymatic activity increased by 26.84% (Figure S5c), which correlates with the lowest pH range from all experiments. Hence, by modulating the pH, we generated an optimal environment that improved enzymatic catalysis [45, 46]. Nevertheless, in this case, we did observe an abrupt decay of the speed of the micromotors, already starting from the lowest HEPES concentration (0.1 mM) (Figure S5a-c and Movie 7). This simultaneous reduction in speed and increase in enzymatic catalysis suggest that the ionic species are physically affecting self-propulsion despite improving enzymatic activity.

### 2.5. Urease Micromotors in NaCl

**Figure 4:**
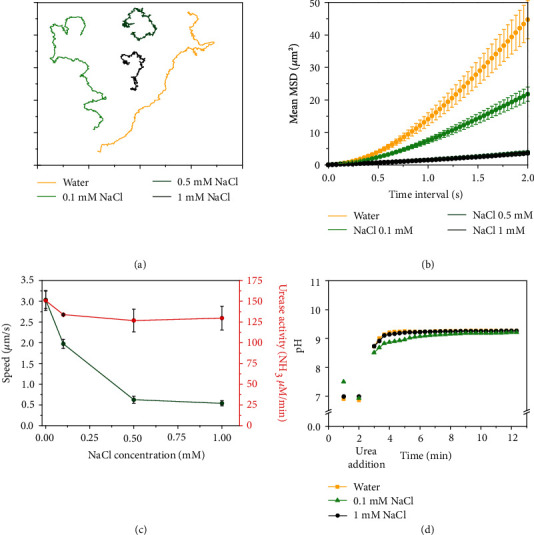
Motion dynamics, enzymatic activity, and pH change produced by urease micromotors for different concentrations of NaCl at 200 mM urea. (a) Representative trajectories (axis divided in 5 *μ*m). (b) Average micromotor MSDs. (c) Average speed and enzymatic activity of micromotors. In (b, c), results are shown as the mean ± standard error of the mean. (d) pH change produced by micromotors.

To rule out the effect of pH on enzymatic activity and to only study the effect of ionic species, we exposed micromotors to NaCl, which is a pH-neutral salt and the most abundant compound in PBS. A similar trend in speed reduction was observed between the basic and the neutral salt (Figures 3(a)–3(c) vs. Figures 4(a)–4(c)). We observed pH changes from 7 up to 9 in all studied conditions (Figure 4(d)). Noteworthily, even though enzymatic activity was maintained upon NaCl addition (Figure 4(c), red), speed was dramatically reduced over the same range of salt concentration (Movie 8), indicating that the motion hampering was not a consequence of enzyme inhibition but caused by the presence of ionic species. From this set of experiments, one can conclude that the presence of ionic species clearly affects the motion of enzymatic micromotors regardless of enzyme activity and pH.

### 2.6. 3D Tracking of Urease Micromotors

**Figure 5:**
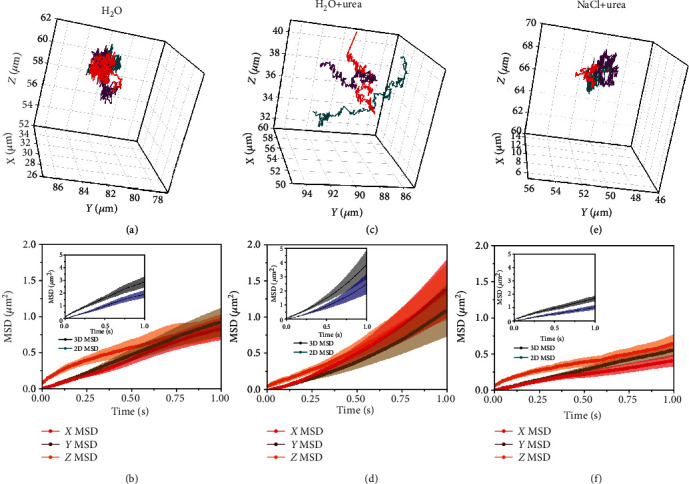
Analysis of 3D motion of urease micromotors in ionic media after 5 minutes of adding urea and/or NaCl. (a) 3D trajectories of three urease micromotors and (b) MSDs from the *X*, *Y*, and *Z* dimensions of urease micromotors in water. (c) 3D trajectories of three urease micromotors and (d) MSDs from the *X*, *Y*, and *Z* dimensions of urease micromotors in water with 200 mM urea. (e) 3D trajectories of three urease micromotors and (f) MSDs from the *X*, *Y*, and *Z* dimensions of urease micromotors in 1 mM NaCl with 200 mM urea. Insets: 2D and 3D MSDs of urease micromotors in (b) water, (d) water with 200 mM urea, and (f) 1 mM NaCl with 200 mM urea.

We studied the 3D motion of micromotors with digital holographic microscopy to analyze the distance of micromotors from the bottom surface and rule out the effect of the bottom surface on the motion. Surfaces have been observed to determine the orientation and have been used as guidance of Pt-based micromotors [47–50]. Moreover, this technique allows us to track micromotors taking into account the *Z* coordinate, providing more accurate results on motion dynamics than *X*‐*Y* tracking.  Figure 5(a) displays several tracking trajectories using this technique, showing Brownian motion of the micromotors without fuel in the three dimensions of space. By calculating the MSDs of each coordinate separately (namely, *X*, *Y*, and *Z*), we could observe that there was no preferred direction of motion (Figure 5(b)). Since the one-dimensional MSDs overlap, we can conclude that the diffusive motion is analogous in all directions. The *Z* coordinate does not show saturation at long times (a sign of constrained motion due to attractions with the bottom surface) or a parabolic shape (a sign of the particle being attracted towards the bottom with a certain speed, e.g., gravity or electrostatic interactions), and we can ensure that the particles were behaving analogously in all three directions of space. In the inset, the MSD in 3D and its 2D projection (using only the *X*‐*Y* coordinates to simulate the optical microscopy experiments from the previous sections, where the *Z* coordinate cannot be taken into account) effectively show the Brownian diffusion of the micromotors without fuel.

Then, as it was first observed in 2D in the previous figures, we confirm how urease micromotors overcome Brownian motion when urea is present in H_2_O and self-propel in *X*, *Y*, and *Z* (Figure 5(c)). In this case, the one-dimensional MSDs also overlap with each other with a quadratic trend, indicating no difference in propulsion among the three directions of space (Figure 5(d)).

The motion behavior returns to Brownian diffusion when adding 1 mM NaCl (Figures 5(e) and 5(f)). There are barely any differences between the one-dimensional MSDs of the cases reported in Figures 5(a) and 5(e), both of which display Brownian motion. We attribute the increased values at very short times of the *Z* MSD (which disappear at longer times) to errors related to the higher uncertainty in the *z*localization of the micromotors due to inherent limitations of the technique and the type of particle. Given these results, the sedimentation (attraction to the glass bottom surface) can be discarded as the cause of motion decay of motors when 1 mM NaCl is added (Figure 5(d)). Additionally, during the digital holographic microscopy experiments, we calculated the particle distance from the bottom surface for all conditions: at 52 ± 10 *μ*m in H_2_O (Figure 5(a)), at 38 ± 15 *μ*m after addition of urea (Figure 5(c)) and at 60 ± 12 *μ*m in 1 mM NaCl with urea (Figure 5(e)). Hence, we ensured that no physical interaction was interfering with self-propulsion.

### 2.7. mPEG-Coated Urease Micromotors in Ionic Media

Since ionic species are ubiquitous in physiological environments, overcoming the motion restriction by ions is of crucial relevance for future biomedical applications. A recent study reported that the ionic tolerance in self-electrokinetic micromotors could be improved through increasing surface conductivity with a polyelectrolyte coating. This coating method also showed promising results for electrolyte self-diffusiophoretic catalytic motors such as SiO_2_/Pt and TiO_2_/Pt Janus nanomotors [41]. Furthermore, the Somasundar et al. group recently reported that by modifying the surface charge of liposome-based enzyme-powered nanomotors, speed and directionality could be tuned. In this case, the presence of ionic species in the solution was also determined to crucially influence the motion behavior [15].

**Figure 6:**
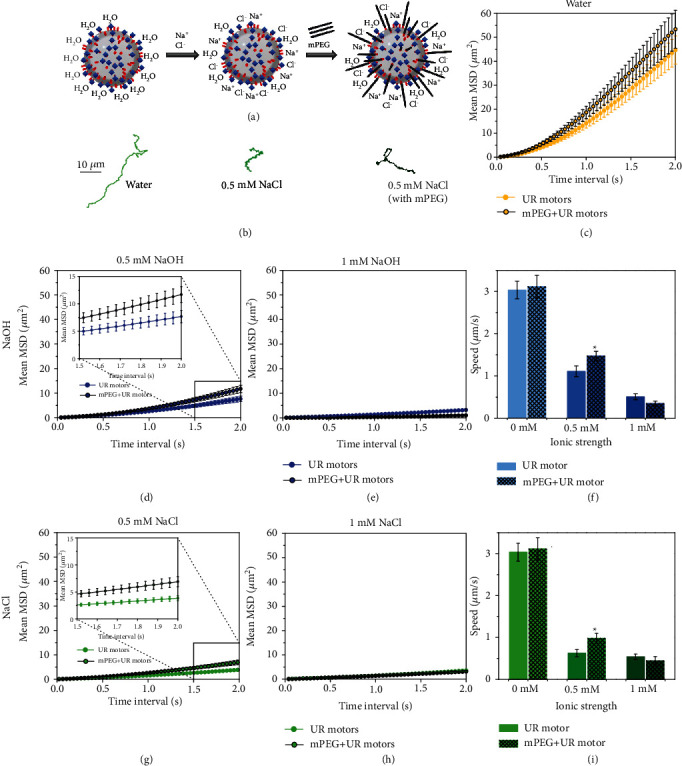
Motion dynamics of urease micromotors with and without methoxypolyethylene glycol amine (mPEG) coating for different concentrations of NaOH and NaCl at 200 mM urea. (a) Schematic of the mPEG coating effect on urease micromotors. (b) Representative trajectories of urease micromotors in water and 0.5 mM NaCl with and without mPEG coating. (c) Mean squared displacement of urease micromotors with and without mPEG 5000 MW in water. Mean squared displacement of urease micromotors with and without mPEG 5000 MW in (d) 0.5 and (e) 1 mM NaOH. (f) Speed of urease micromotors with and without mPEG 5000 MW in different NaOH concentrations. Mean square displacement of urease micromotors with and without mPEG 5000 MW in (g) 0.5 and (h) 1 mM NaCl. (i) Speed of urease micromotors with and without mPEG 5000 MW in different NaCl concentrations. Results are shown as the mean ± standard error of the mean, where “∗” denotes significant differences compared to the control group (*p* < 0.05, *N* = 30).

Under this framework, we attempted to improve the ionic tolerance of enzyme-powered micromotors by modifying their surface with a polyethylene glycol-based coating (Figures 6(a) and 6(b)). Both urease and a heterobifunctional mPEG were incubated together and attached to the GA linker through their respective amino groups. The mPEG coating increased the zeta potential compared to the bare urease micromotors (Figure S6) from −10.1 ± 1.7 mV to −31.9 ± 2.1 mV and mildly increased the hydrodynamic radius (Figure S7). While the bare urease micromotors had the zeta potential dramatically decreased when increasing pH (adding NaOH or producing NH_3_ from urea catalysis), the mPEG coating softens these changes in zeta potential (Figure S8). The presence of mPEG chains did not affect the motion capabilities in water (Figure 6(c)). Interestingly, we observed that mPEG significantly (*p* < 0.05, *N* = 30) improved micromotor speed at intermediate concentrations (0.5 mM) by 30.7% and 57.0% of both NaOH (Figures 6(d)–6(f)) and NaCl (Figures 6(g)–6(i)) salts, respectively. However, at higher ionic strength (1 mM), the speed decreased regardless of mPEG coating. A similar improvement in motion was observed at 0.004x PBS when coating urease micromotors with mPEG (Figure S9a). In the case of HEPES,the mPEG coating did not affect micromotor speed (Figure S9b).

## 3. Discussion

The effect of ionic media on the self-propulsion capabilities of micromotors could be explained by two different ion-dependent mechanisms: (i) the self-electrokinetic mechanism and (ii) the ionic self-diffusiophoretic mechanism.

The self-electrokinetic mechanism can be ruled out for urease silica-based micromotors because (i) silica has low conductivity and (ii) there are no redox reactions taking place on the surface; therefore, there is no electron flow between different regions of the particle. Ionic self-diffusiophoresis is a more plausible explanation because urease decomposes urea into ionic species (CH_4_N_2_O + 2H_2_O⟶2NH_4_^+^ + CO_3_^2−^) of different diffusivities (NH_4_^+^: 1.957 × 10^−5^ cm^2^ s^−1^ and CO_3_^2-^: 0.923 × 10^−5^ cm^2^ s^−1^) [51] and thus generates an ionic gradient. The generation of a cationic and anionic gradient can lead to (i) the generation of local electric fields by electrolyte gradients and (ii) a fluid flow from areas of higher ionic concentration to areas of lower ionic concentration. The diffusiophoretic flow that drives propulsion is a result of combining these electrophoretic and chemophoretic effects [52].

Both ionic self-diffusiophoresis and self-electrokinetic mechanisms are based on the generation of electric fields, and the motion is driven by Coulomb interaction between the charges of the surface and the Debye layer [53]. The Debye layer is dramatically reduced by the presence of ionic media; hence, the observed effect of external ions on self-propulsion could be explained by a hindrance of the product gradients, thus reducing motion capabilities.

A recent theoretical model correlates the reduction of speed of urease micromotors with the concentration of electrolytes in their surroundings, predicting a decrease of velocity at increasing salt concentrations, caused by a reduction of the Debye length. This model also takes into account the relevance of the particle charge for self-propulsion, crucial in the working ionic strengths [54]. Hence, by coating urease micromotors with mPEG and decreasing surface charge, we may be generating a shielding effect that reduces the influence of the surrounding ions and enhances the gradient of ionic products. This is supported by the mild effect of basic species on the zeta potential once they are coated with mPEG, compared to the bare urease micromotors.

Tuning surface charge has been recently reported to increase the ion tolerance of self-electrokinetic micromotors [41]. Similarly, the fact that mPEG increases the negative zeta potential of the particle indicates a higher electrophoretic mobility, and this could open the possibility that mPEG is promoting the electrophoretic effect of ionic self-diffusiophoresis. However, there is only a slight increase in motion (without salts) after coating with mPEG, and we observe no clear correlation between the zeta potential and the speed. Since motion is reduced although the zeta potential is increased, this could be explained by the fact that micromotors require a significant ionic gradient around the particles in order to achieve motion. Hence, it is plausible that the mPEG coating contributes to the chemophoretic effect by maintaining this ionic gradient and avoiding the reduction in the Debye length forced by external ions.

Moreover, a more negative charge on the surface has been suggested to locally acidify the microenvironment, which would improve urease catalysis [45, 46]. However, we did not find a clear correlation between the enzyme activity and motion performance, and in the case of HEPES, enzyme activity was always maintained or even increased with more ionic strength, while the speed decreased. For this reason, if there is such local acidification, there may be a limited contribution to ion tolerance, and further research is needed to fully confirm its effect.

This surface charge modification and recovery of the Debye length of urease micromotors in ionic media pave the way towards overcoming motion limitations in physiological environments.

In summary, we assessed the self-propulsion of urease micromotors in different ionic media, showing that the presence of ionic species negatively affects their motion capabilities, regardless of enzymatic activity and pH. We discard any physical interaction with the surface as the cause of motion decay by analyzing the 3D navigation of urease micromotors using holographic microscopy. To circumvent this issue, we modified the micromotors' surface charge with a mPEG coating, which resulted in a motion improvement for 0.5 mM ionic strength for both NaCl and NaOH. The characteristics of our system and the interference of ions in the motion capabilities point towards an ionic self-diffusiophoretic mechanism. This work paves the way towards not only understanding the dynamics of enzyme-powered motors at a fundamental level but also considering their promising biomedical applications.

## 4. Materials and Methods

### 4.1. Materials and Chemicals

The following are the materials and chemicals: 2 *μ*m microparticles based on polystyrene (Sigma-Aldrich cat. no. 78452), ethanol 99% (PanReac AppliChem cat. no. 131086-1214), ammonium hydroxide solution (Sigma-Aldrich cat. no. 221228), 3-aminopropyltriethoxysilane (APTES) 99% (Sigma-Aldrich cat. no. 440140), tetraethylorthosilicate (TEOS) ≥ 99% (Sigma-Aldrich cat. no. 86578), dimethylformamide (DMF) ≥ 99.8% (Acros Organics cat. no. 423640010), 1x phosphate-buffered saline (PBS) (Thermo Fisher Scientific cat. no. 70011-036), glutaraldehyde (GA) (25 wt%) (Sigma-Aldrich cat. no. G6257), urease from *Canavalia ensiformis* (Jack bean) (Sigma-Aldrich cat. no. U4002), methoxypolyethylene glycol amine 5000 MW (mPEG-5k) (Sigma-Aldrich, Catalog No. 06679), Krypton™ protein staining (Thermo Scientific cat. no. 46628), Pierce™ BCA Protein Assay Kit (Thermo Fisher cat. no. 23227), Urease Activity Assay Kit (Sigma-Aldrich cat. no. MAK120), urea (Sigma-Aldrich cat. no. U5128), sodium chloride (NaCl) (Sigma-Aldrich, Catalog No. 31434-1KG-R), sodium hydroxide (NaOH) (Sigma-Aldrich, Catalog No. 30620-1KG-M), and 4-(2-hydroxyethyl)-1-piperazineethanesulfonic acid (HEPES) (Thermo Fisher, Catalog No. 15630056). SEM images were captured by a FEI NOVA NanoSEM 230. TEM images were captured by a Zeiss EM 912. The diameter and zeta potential measurements were performed with a Zetasizer Nano S from Malvern Panalytical. The optical videos and the fluorescent images of the urease micromotors were recorded using the camera (Hamamatsu Digital Camera C11440) of an inverted optical microscope (Leica DMi8). The absorbance measurements were done with the Benchmark Plus Microplate reader from Bio-Rad. The pH measurements were performed with pH meter GLP 21 from Crison Instruments. Silicon spacer of 250 *μ*m is from Grace Bio-Labs CWS-S-0.25, diode laser (635 nm) is from Lasertack LDM-635-200-c,1 × 2optical fiber splitter is from Thorlabs TN632R5F1, 40x/0.65 NA objective is from Olympus PLANFL40X, CMOS camera is from Basler acA1920-155um (5.86 *μ*m × 5.86 *μ*mpixel size), 250 mm lens is from Thorlabs AC508-250-A, and 90 : 10 beam splitter is from Thorlabs BSX16.

### 4.2. Synthesis of Hollow Silica Microcapsules

The silica capsules were synthesized by mixing 250 *μ*l of 2 *μ*m particles based on polystyrene (PS) (Sigma-Aldrich cat. no. 78452), 0.5 ml ethanol 99% (PanReac AppliChem cat. no. 131086-1214), and 0.4 ml ultrapure water. Next, 25 *μ*l ammonium hydroxide solution (Sigma-Aldrich cat. no. 221228) was added, and the mixture was magnetically stirred for 5 min. Then, 2.5 *μ*l 3-aminopropyltriethoxysilane (APTES) 99% (Sigma-Aldrich cat. no. 440140) was added, and the reaction was left to proceed for 6 hours. After, 7.5 *μ*l tetraethylorthosilicate (TEOS) ≥ 99% (Sigma-Aldrich cat. no. 86578) was added to the solution to react overnight. Next, the PS beads coated with a silica shell were washed with ethanol 3 times. The PS was then removed with 4 washes of dimethylformamide (DMF) ≥ 99.8% (Acros Organics cat. no. 423640010). Afterwards, the obtained hollow silica microcapsules were washed 3 more times with ethanol 99% and stored at room temperature.

### 4.3. Functionalization of Silica Capsules with Enzymes

To fabricate urease hollow silica micromotors, the silica capsules were washed 3 times with ultrapure water and 1 time with 1x phosphate-buffered saline (PBS) (Thermo Fisher Scientific cat. no. 70011-036). Then, the particles were suspended in 1x PBS containing glutaraldehyde (GA) (2.5 wt%) (Sigma-Aldrich cat. no. G6257) and kept at room temperature mixing for 3 h. Next, the silica capsules functionalized with GA were washed 3 times with 1x PBS and resuspended again in 1x PBS (pH = 7.4) with 3 mg ml^−1^ of urease from *Canavalia ensiformis* (Jack bean) (Sigma-Aldrich cat. no. U4002) and 4 mg ml^−1^ of mPEG 5000 MW (Sigma-Aldrich, Catalog No. 06679) when necessary. The solution was left overnight and washed 3 times with 1x PBS. The supernatants discarded in this process were used for the total protein quantification (Figure S2). Then, the solution of urease micromotors in 1x PBS is divided in aliquots and stored at -4°C to be used the same day.

### 4.4. Hydrodynamic Diameter and Zeta Potential Measurements

The hydrodynamic diameter (Figure 1 and Figures S4 and S6) and zeta potential (Figure 1 and Figures S3, S5, and S8) of the microparticles were measured in each step of the functionalization process through a dynamic light scattering (DLS) method. The Zetasizer Nano S from Malvern Panalytical was used for the diameter and zeta potential characterization. For zeta potential measurements, the samples were measured for a minimum of 10 times per condition with a scattering angle of 173° and using the Henry equation. For size estimation, 15 analyses were performed per condition with a scattering angle of 12.8°.

### 4.5. Total Protein Quantification

The presence of enzyme attached on the surface of the silica capsules was confirmed indirectly through a total protein quantification of the supernatants discarded on the 1x PBS washes of the functionalization process (Figure S2). The protocols *Preparation of Standards and Working Reagents* and *Microplate Procedure* (*Sample* to *WR* *ratio* = 1 : 8) were followed to perform the total protein quantification as specified in the document of Instructions of the Pierce™ BCA Protein Assay Kit (Thermo Fisher cat. no. 23227). By these means, the protein quantified in the supernatants discarded after functionalization was subtracted to the protein quantity contained in the enzyme powder.

### 4.6. Fluorescent Krypton™ Protein Labelling

The presence of enzyme on the microparticle surface was confirmed (Figure S1) through a Krypton™ protein staining assay (Thermo Fisher Scientific, Catalog No. 46628). The Krypton™ protein staining was diluted 10-fold with ultrapure water and used to resuspend the microparticles at different stages of the functionalization. The solution was mixed in an orbital shaker for 20 minutes. After 2 washes with ultrapure water, the sample was observed under a microscope and fluorescent images were taken using a Hamamatsu Digital Camera (C11440). For this fluorescent dye, the wavelength of excitation is 520 nm and the detection wavelength was 580 nm. Dark conditions were needed during the entire preparation of the sample. An untreated control condition of microparticles with GA was also done to check glutaraldehyde autofluorescence.

### 4.7. Optical Video Recording

The motion of the urease micromotors was recorded using the camera (Hamamatsu Digital Camera C11440) of an inverted optical microscope (Leica DMi8). The 63x water immersion objective was used to record the micromotors placed on a glass slide, thoroughly mixed with the specific solutions containing 200 mM urea, selected to cover the range at which these enzymes are active and show the Michaelis-Menten growth kinetics, as reported in BRENDA, the Comprehensive Enzyme Information System (https://www.brenda-enzymes.org/). The glass slide was covered with a coverslip, and videos of 25 FPS and 30-35 seconds were recorded up to the first 3 min after mixing. At least, 20 urease micromotors were recorded for each different condition.

### 4.8. Data Analysis of Motion

The videos were analyzed using custom-designed tracking Python software to obtain the tracking trajectories of the microparticle displacement. From the *X* and *Y* values over time, the MSD was calculated using

(1) $$\begin{array}{c} \text{MSD}\left(\Delta t\right)=<\overset{n}{\underset{i}{\sum }}{\left(r_{i}\left(t+\Delta t\right)-r_{i}\left(t\right)\right)}^{2}>, \end{array}$$

where *t* is the time, *r*_*i*_(*t*) is the position of the particle in the coordinate *i* at time *t*, *n* = 2 are the dimensions of 2D analysis, and <·> denotes ensemble and time average. The velocity (*v*) was then extracted from fitting the MSD to

(2) $$\begin{array}{c} \text{MSD}\left(\Delta t\right)=4D_{t}t+v^{2}t^{2}, \end{array}$$

where *D*_*t*_ is the diffusion coefficient and *v* is the speed, since we analyze the propulsive regime when *t* ≪ *τ*_*r*_, being *τ*_*r*_ the rotational diffusion time and *t* the time of MSD represented [55]. *τ*_*r*_ was calculated to be 5.579 ± 0.018 s, which is

(3) $$\begin{array}{c} \tau_{r}=\frac{1}{D_{r}}, \end{array}$$

where *D*_*r*_ is the rotational diffusion coefficient (*D*_*r*_ = 0.1792 ± 0.0006 s^−1^), which depends on the radius of the particle, as it can be observed in the Stokes-Einstein equation:

(4) $$\begin{array}{c} D_{r}=\frac{k_{B}T}{8\pi \eta r^{3}}, \end{array}$$

where *k*_*B*_ is the Boltzmann constant, *T* is the absolute temperature, *η* is the solvent viscosity, and *r* is the radius of the diffusing particle. Hence, *τ*_*r*_ depends on the temperature (*T* = 24 ± 1°C), the solvent viscosity (*η* = 0.9107 · 10^−3^ kg m^−1^ s^−1^), and the radius of the particle (*r* = 1.00 ± 0.05 *μ*m).

The statistical analysis presented in the intermediate concentrations (0.5 mM) of NaCl and NaOH in Figures 5(c) and 5(d), respectively, compares the effect of mPEG-5k MW coating on speed and is done by applying an unpaired *t*-test, both showing a *p* value < 0.05: NaCl: *p* < 0.0177 and NaOH: *p* < 0.0327.

### 4.9. Urease Enzymatic Activity

The enzymatic activity of urease attached to silica capsules was evaluated for all the different conditions (Figures 2–5) using the Urease Activity Assay Kit (Sigma-Aldrich cat. no. MAK120) based on the Berthelot method [44]. The process followed is detailed in the *Technical Bulletin of the Urease Activity Assay Kit*. It works through the ammonia generated (Equation (5)) obtained in the catalysis of urea (Sigma-Aldrich cat. no. U5128) by urease and monitoring the absorbance at 670 nm [44]. The enzymatic reaction is the following:

(5) $$\begin{array}{c} \text{urea}\overset{\text{urease}}{⟶}2{\text{NH}}_{3}+{\text{CO}}_{2}. \end{array}$$

The enzymatic activity is investigated for 3 minutes in the different conditions, by incubating the urease micromotors in the different conditions with the urea provided in the kit.

### 4.10. pH Measurements

The pH measurements were performed in 1.5 ml tubes using the pH meter GLP 21 from Crison Instruments. After two initial pH readings of the solution containing the urease micromotors in every experimental condition (Figures 2–5), urea was added at minute 2.5 to end up with a 200 mM urea solution. Then, the pH was measured every 20 seconds from minute 3 to minute 12 to detect the evolution over time.

### 4.11. Sample Chambers for Digital Holographic Microscopy

For the sample chambers, firstly, #1.5 glass coverslips were cleaned by sequential bath sonication in ultrapure water, ethanol, isopropanol, and ultrapure water for 10 minutes each. Subsequently, the glass coverslips were incubated with a solution of 10 mg ml^−1^ bovine-serum-albumin for 30 minutes prior to rinsing with ultrapure water to passivate the surface and prevent nonspecific binding of the micromotors to the surface. Next, a self-adhering silicon spacer of 250 *μ*m (Grace Bio-Labs CWS-S-0.25) was placed onto one side of the glass substrate, upon which the respective solutions of micromotors were added: (i) water, (ii) water with 200 mM urea, and (iii) water with 1 mM NaCl and 200 mM urea. Finally, the chamber was gently sealed by pressing an additional functionalized glass coverslip on top. Immediately upon assembly, the sample chamber was placed on the digital holographic microscope and imaged for the following 10 minutes.

### 4.12. 3D Motion Recording and Analysis with Digital Holographic Microscopy

The 3D motion of the micromotors was recorded on a custom-built off-axis digital holographic microscope operating in transmission with Koehler illumination. In detail, light from a 635 nm diode laser (Lasertack LDM-635-200-c) was split into an imaging and reference arm using a 1 × 2 optical fiber splitter (Thorlabs TN632R5F1). Light from the imaging arm, impinging on the sample chamber, was collected by a 40x/0.65 NA objective (Olympus PLANFL40X) and focused onto a CMOS camera (Basler acA1920-155um, 5.86 *μ*m × 5.86 *μ*m pixel size) with a 250 mm lens (Thorlabs AC508-250-A), giving an effective 55x magnification. To generate the hologram, a 90 : 10 beam splitter (Thorlabs BSX16), positioned after the 250 mm tube lens, was used to recombine and interfere the imaging and reference arms at the plane of the camera chip.

All 3D tracking experiments were performed by acquiring 1000 holograms at a frame rate of 100 Hz. To minimize light exposure, the sample was only irradiated for 180 *μ*s, i.e., the duration of the camera exposure. Similarly, the fluence on the sample was between 10^−6^ and 10^−5^ mW/*μ*m^2^, as determined by measuring the power of the laser at the sample as well as the illumination area: 100 *μ*W and circular area with a diameter of 200 *μ*m, respectively.

The recorded off-axis holograms were first processed by Fourier filtering to extract the corresponding amplitude and phase image from the interference term. Succinctly, the hologram was Fourier transformed, whereby three nonoverlapping regions were identified as the real, twin, and zero-order image in *k*-space, respectively. The real image, one of the interference terms, was then isolated by hard-aperture selection and frequency demodulated by shifting its position in *k*-space to the zero-frequency. To complete the Fourier filtering process, an inverse Fourier transform was performed on the demodulated and isolated real image in *k*-space. Next, a background hologram, containing all static features in the sample, was generated by taking the median pixel-wise over all the processed holograms in a single acquisition. Subtraction of the processed holograms by the background hologram resulted in the analysis and tracking of only dynamic features in the sample. To enhance the detection of micromotors, a digital aperture mask with a radius of 5 pixels was placed at the center of the *k*-space of each processed hologram, analogous to darkfield-based detection. Then, the resulting holograms were propagated along the optical axis, from -10 *μ*m to +110 *μ*m with a spacing Δ*z* = 0.5 *μ*m, according to the angular spectrum method to produce 3D amplitude maps [56]. Briefly, the processed *NxN* holograms were convolved with a propagation kernel of the form

(6) $$\begin{array}{c} K\left(x,y,z\right)=\exp\left(iz\sqrt{{k_{m}}^{2}-{k_{x}}^{2}-{k_{y}}^{2}}\right), \end{array}$$

where *k*_*m*_ = 2*nπ*/*λ*, with *n* being the refractive index of medium where the propagation is performed, water. The discretized spatial frequencies are (*k*_*x*_, *k*_*y*_) = 2*π*(*x*, *y*)/*n*Δ*x* for (−*N*/2 ≤ *x*, *y* < *N*/2) and with Δ*x* representing the magnified pixel size of the imaging system. For 3D localization, regions of interest containing possible candidate particles were identified by finding local maxima in the 3D amplitude maps. To achieve subpixel localization in the *X*‐*Y* coordinates within the identified regions of interest, micromotors that were in focus at a specific *Z* plane were fitted by a 2D Gaussian. For *Z* coordinate localization, sub-Δ*Z* sampling was determined by first calculating the Tamura values ($T\left(z\right)=\sqrt{\sigma \left(lz\right)/\text{mean}\left(lz\right)}$ for a region of interest of (≈2 × 2 *μ*m^2^) centered about the intensity maxima for each *Z* plane and then fitting a parabola using the three most adjacent pixel values along the maximum. The localization along the *Z* axis (≈200 nm) was significantly worse than along either the *X* or *Y* axis (≈5 nm) due to characteristic point spread function of the micromotors, which led to multiple local maxima. We attribute this larger uncertainty in localization along the *Z* axis specifically to the lack of a model to capture the scattering response for these specific microparticles that are larger than the diffraction limit and hollow. The resulting 3D localizations were linked according to the algorithm of Jaqaman et al. [57] of which only those with more than 250 time points were used for further analysis.

### 4.13. Computation of MSDs from 3D-Tracked Data

The trajectories as (*X*, *Y*, *Z*) coordinates provided by the 3D motion recording with digital holographic microscopy were used to compute the MSD of such trajectories in different ways, in order to understand possible deviations from the active particle theory of motion upon addition of fuel or salts, as reported in Figure 5.

Firstly, the MSD in 3D was computed as

(7) $$\begin{array}{c} 3\text{D MSD}\left(\Delta t\right)=<\overset{3}{\underset{i}{\sum }}{\left(r_{i}\left(t+\Delta t\right)-r_{i}\left(t\right)\right)}^{2}>, \end{array}$$

considering the three dimensions of space, where *r*_*i*_(*t*) is the position of the particle in the coordinate *i* and moment *t* of time. The 2D MSD was computed assuming a projection of the trajectory onto the *XY* plane, that is, only considering *X* and *Y* coordinates for the analysis and not the *Z* component. This is analogous to the results presented in the previous figures and acquired by optical microscopy, in which one has only access to *X* and *Y*. This calculation is analogous to that of Equation (1). The 2D MSD should, in theory, present the same shape as the full 3D MSD, as proven in Figure 5.

The 1D MSDs in *X*, *Y*, or *Z* were simply computed as projections of the trajectories onto one of the three dimensions of space, obtaining

(8) $$\begin{array}{c} X\text{ MSD}\left(\Delta t\right)=<{\left(x\left(t+\Delta t\right)-x\left(t\right)\right)}^{2}>,\\ Y\text{ MSD}\left(\Delta t\right)=<{\left(y\left(t+\Delta t\right)-y\left(t\right)\right)}^{2}>,\\ Z\text{ MSD}\left(\Delta t\right)=<{\left(z\left(t+\Delta t\right)-z\left(t\right)\right)}^{2}>, \end{array}$$

where *x*, *y*, and *z* are the three coordinates of space and <·> denotes ensemble and time average. Theoretically, all three MSDs should follow the same trend (namely, diffusive or propulsive behavior), since there is no preferred direction in space. However, if there was confinement in the *Z*-direction, due to the addition of salts, or sedimentation with a finite speed, the *Z* MSD should be different from the other two.

## Data Availability

All data is available in the main text or in Supplementary Materials.

## Supplementary Materials

Supplementary Materials Figure S1: Krypton™ protein staining dye before and after functionalization of micromotors. Figure S2: total protein quantification of unattached enzyme after functionalization. Figure S3: zeta potential measurements of the urease micromotor fabrication process. Figure S4: complete spectrum of the diameter measurements of silica microparticles for each step of the functionalization process. Figure S5: motion dynamics, enzymatic activity, and pH change produced by urease micromotors for different concentrations of HEPES at 200 mM urea. Figure S6: zeta potential measurements of the mPEG-coated urease micromotor fabrication process. Figure S7: diameter measurements of urease micromotors with and without mPEG coating. Figure S8: zeta potential of urease micromotors in different ionic environments. Figure S9: mean squared displacement of urease micromotors with and without methoxypolyethylene glycol amine (mPEG) coating in PBS, HEPES, NaOH, and NaCl. Movie 1: micromotor recording over 270 *μ*m of *Z* coordinate in H_2_O (AVI). Movie 2: micromotor recording over 270 *μ*m of *Z* coordinate in H_2_O with 200 mM urea (AVI). Movie 3: micromotor recording over 270 *μ*m of *Z* coordinate in 1x PBS (AVI). Movie 4: micromotor recording over 270 *μ*m of *Z* coordinate in 1x PBS with 200 mM urea (AVI). Movie 5: micromotor self-propulsion at 200 mM urea for different concentrations of PBS (AVI). Movie 6: micromotor self-propulsion at 200 mM urea for different concentrations of NaOH (AVI). Movie 7: micromotor self-propulsion at 200 mM urea for different concentrations of HEPES (AVI). Movie 8: micromotor self-propulsion at 200 mM urea for different concentrations of NaCl (AVI). Movie 9: micromotor Brownian motion at 0 mM urea for different concentrations of PBS, NaOH, NaCl, and HEPES (AVI).
