# Supplementary material for: Ionic Species Affect the Self-Propulsion of Urease-Powered Micromotors
Source: Research (Wash D C). 2020 Jul 27;2020:2424972. doi: 10.34133/2020/2424972 (PMC7404610; doi:10.34133/2020/2424972)
Supplement: Supplementary Materials — Figure S1: Krypton™ protein staining dye before and after functionalization of micromotors. Figure S2: total protein quantification of unattached enzyme after functionalization. Figure S3: zeta potential measurements of the urease micromotor fabrication process. Figure S4: complete spectrum of the diameter measurements of silica microparticles for each step of the functionalization process. Figure S5: motion dynamics, enzymatic activity, and pH change produced by urease micromotors for different concentrations of HEPES at 200 mM urea. Figure S6: zeta potential measurements of the mPEG-coated urease micromotor fabrication process. Figure S7: diameter measurements of urease micromotors with and without mPEG coating. Figure S8: zeta potential of urease micromotors in different ionic environments. Figure S9: mean squared displacement of urease micromotors with and without methoxypolyethylene glycol amine (mPEG) coating in PBS, HEPES, NaOH, and NaCl. Movie 1: micromotor recording over 270 μm of Z coordinate in H2O (AVI). Movie 2: micromotor recording over 270 μm of Z coordinate in H2O with 200 mM urea (AVI). Movie 3: micromotor recording over 270 μm of Z coordinate in 1x PBS (AVI). Movie 4: micromotor recording over 270 μm of Z coordinate in 1x PBS with 200 mM urea (AVI). Movie 5: micromotor self-propulsion at 200 mM urea for different concentrations of PBS (AVI). Movie 6: micromotor self-propulsion at 200 mM urea for different concentrations of NaOH (AVI). Movie 7: micromotor self-propulsion at 200 mM urea for different concentrations of HEPES (AVI). Movie 8: micromotor self-propulsion at 200 mM urea for different concentrations of NaCl (AVI). Movie 9: micromotor Brownian motion at 0 mM urea for different concentrations of PBS, NaOH, NaCl, and HEPES (AVI). [file 2424972.f1.zip › SupplementaryInformation_ions.pdf]

Supplementary Information

Ionic Species Affect the Self-propulsion of Urease-Powered Micromotors

Xavier Arqué, Xavier Andrés, Rafael Mestre, Bernard Ciraulo, Jaime Ortega Arroyo, Romain Quidant, Tania Patiño*, Samuel Sánchez*

*e-mail: tpatino@ibecbarcelona.eu

*e-mail: ssanchez@ibecbarcelona.eu

Supplementary Figures

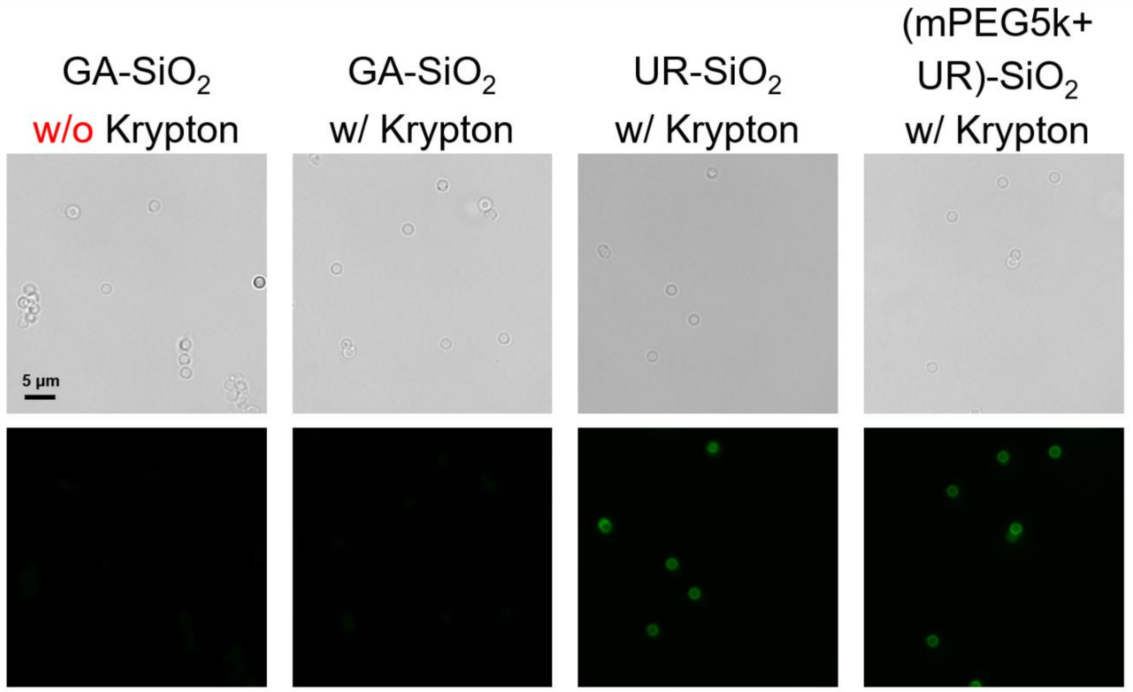

Figure S1. Bright field and fluorescent (Ex/Em = 520/580 nm) images of urease micromotors treated with Krypton™ protein staining dye before and after functionalization with urease and mPEG-5k MW.

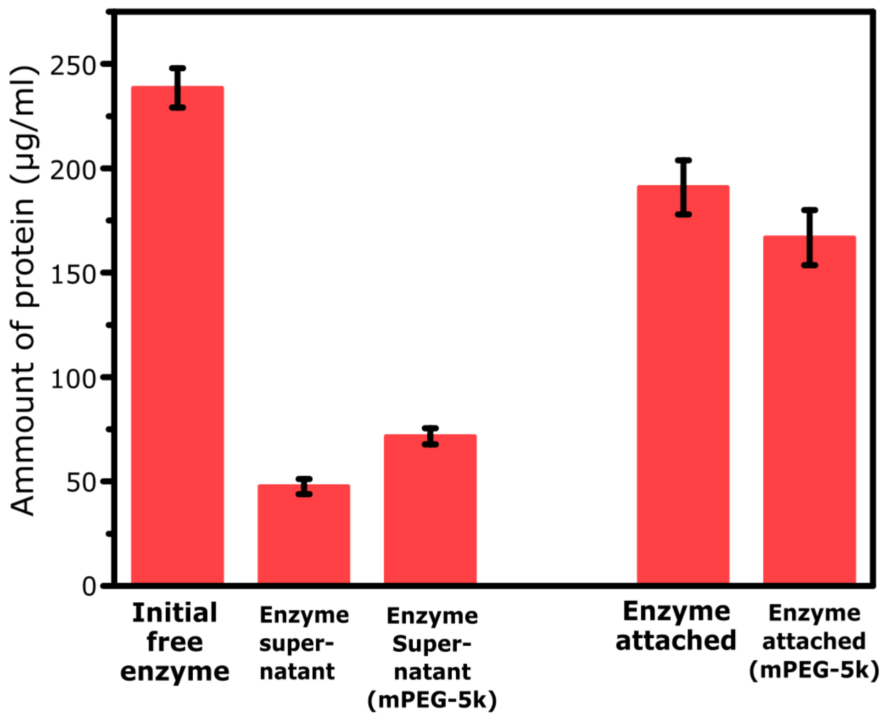

Figure S2. Total protein quantification of free enzyme in the functionalization supernatant, with and without applying a mPEG-5k coating on the micromotors.

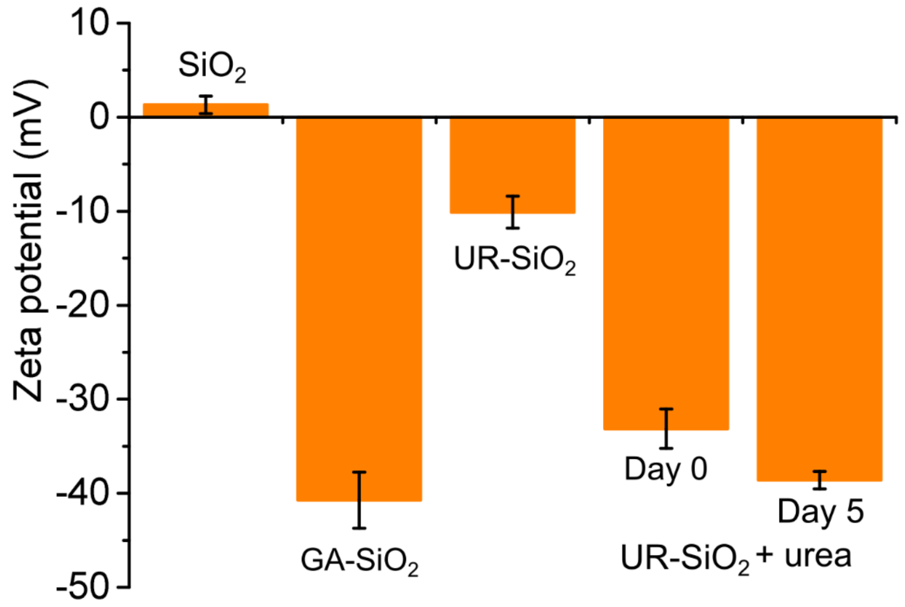

Figure S3. Zeta potential measurements of the urease micromotors fabrication process and in day 0 and 5 after urea addition.

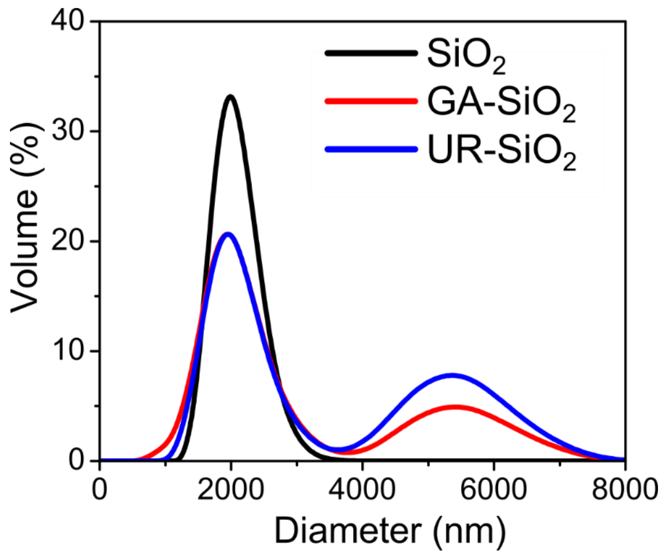

Figure S4. Complete spectrum of the diameter measurements of silica microparticles for each step of the functionalization process. Particles with sizes between 4-7 µm correspond to aggregates, but only single particles (78.9% of the sample) were considered for motion analysis.

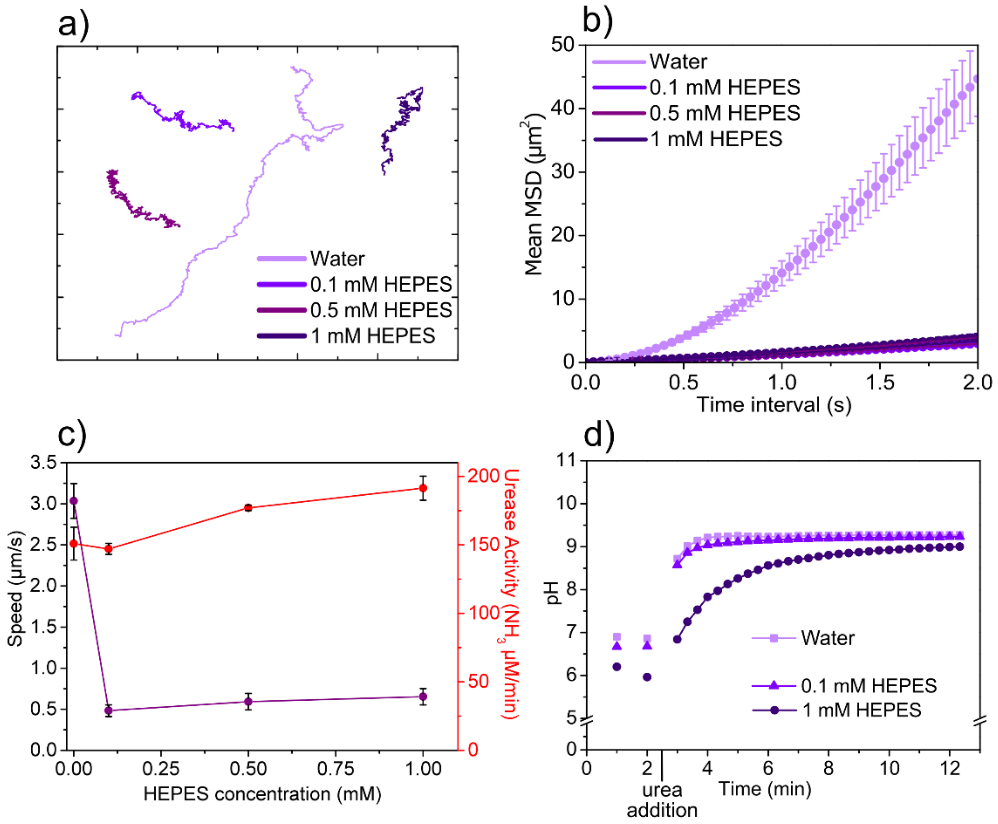

Figure S5. Motion dynamics, enzymatic activity and pH change produced by urease micromotors for different concentrations of HEPES at 200 mM urea. (a) Representative tracking trajectories (axis divided in 5 µm). (b) Average micromotor MSDs. (c) Average speed and enzymatic activity of micromotors. In b and c results are shown as the mean ± standard error of the mean. (d) pH change produced by micromotors.

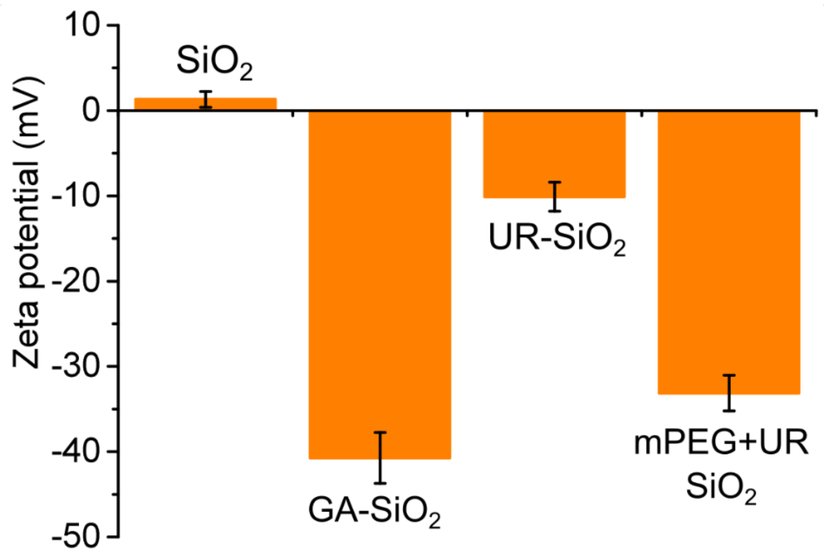

Figure S6. Zeta potential measurements of the mPEG-coated urease micromotors fabrication process.

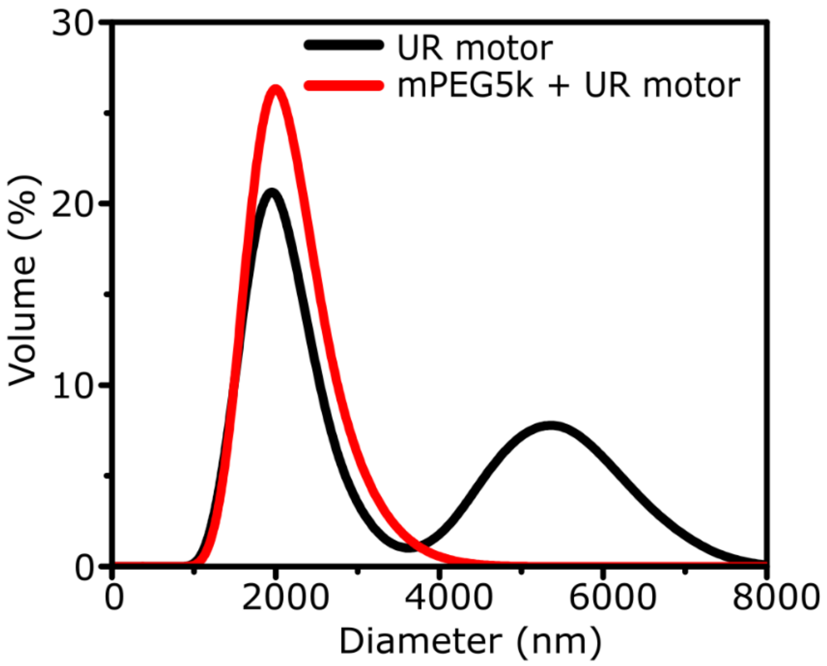

Figure S7. Diameter measurements of urease micromotors with and without mPEG-5k MW coating.

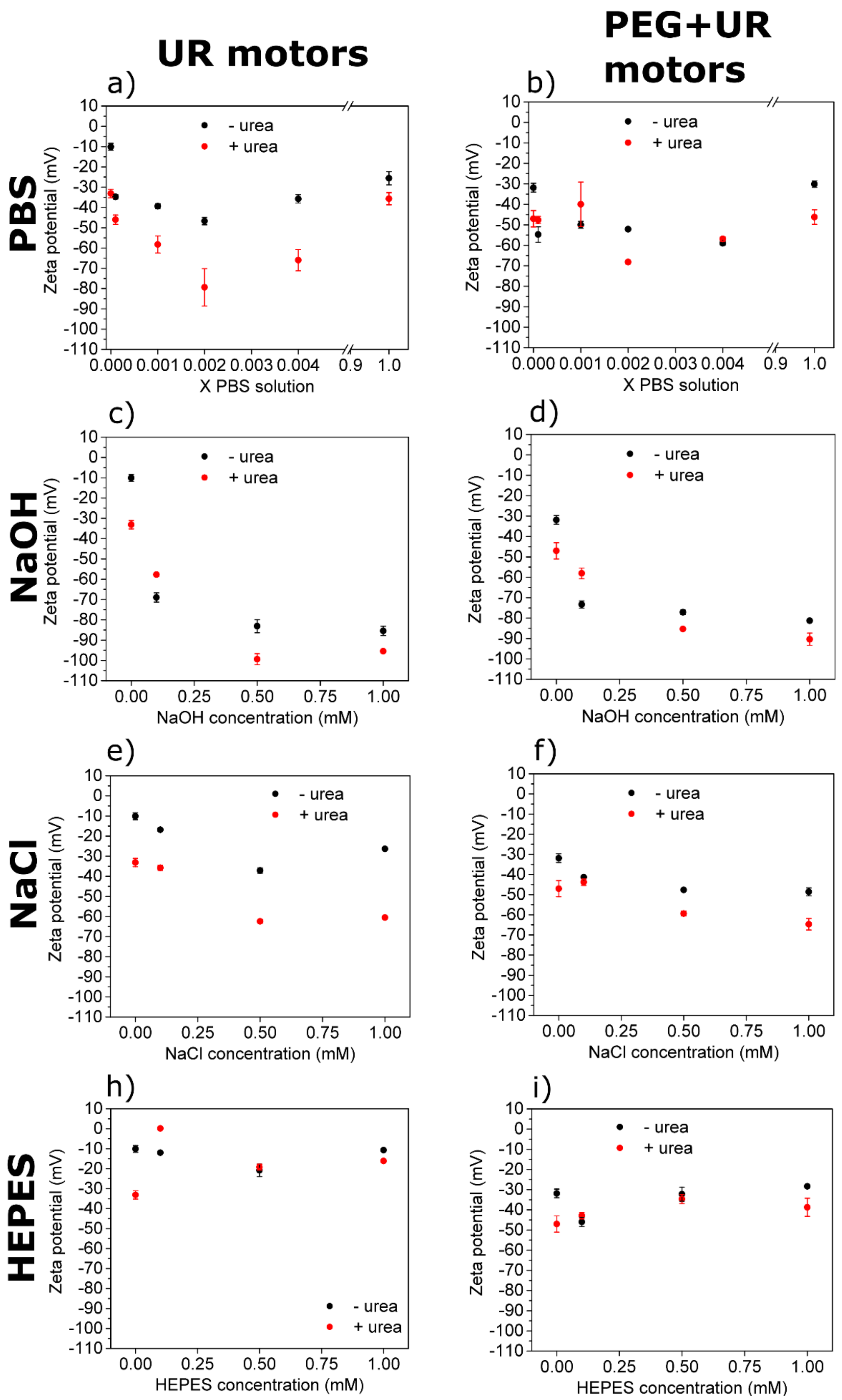

Figure S8. Zeta potential of urease micromotors in different ionic environments. Zeta potential measurements of urease micromotors with and without mPEG coating in different concentrations of (a, b) PBS, (c, d) NaOH, (e, f) NaCl and (h, i) HEPES, before and after adding 200 mM urea.

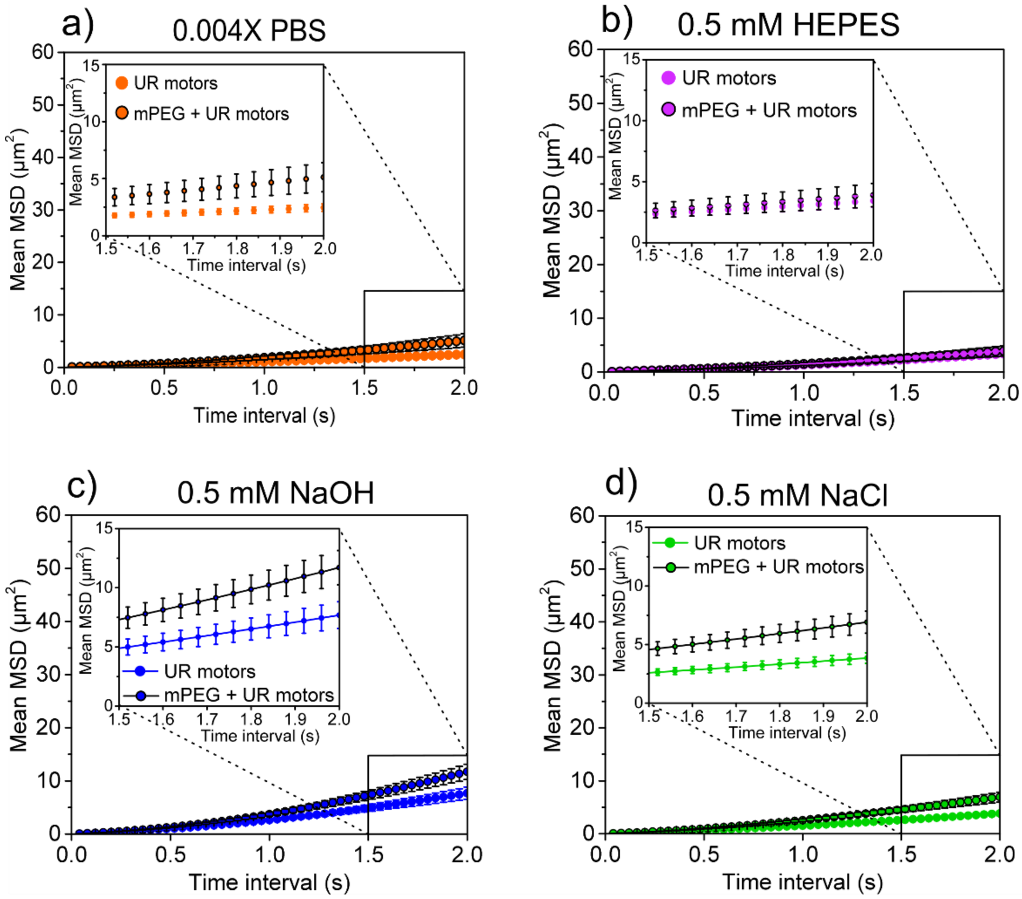

Figure S9. Mean squared displacement of urease micromotors with and without methoxypolyethylene glycol amine (mPEG) coating in PBS, HEPES, NaOH and NaCl at 200 mM urea. (a) Mean squared displacement of urease micromotors with and without mPEG 5000 MW in 0.004X PBS. (b) Mean squared displacement of urease micromotors with and without mPEG 5000 MW in 0.5 mM HEPES. (c) Mean squared displacement of urease micromotors with and without mPEG 5000 MW in 0.5 mM NaOH. (d) Mean squared displacement of urease micromotors with and without mPEG 5000 MW in 0.5 mM NaCl.

Movie 1. Micromotor recording over 270 µm of Z coordinate in H_2O (AVI).

Movie 2. Micromotor recording over 270 µm of Z coordinate in H_2O with 200 mM urea (AVI).

Movie 3. Micromotor recording over 270 µm of Z coordinate in 1X PBS (AVI).

Movie 4. Micromotor recording over 270 µm of Z coordinate in 1X PBS with 200 mM urea (AVI).

Movie 5. Micromotor self-propulsion at 200 mM urea for different concentrations of PBS (AVI).

Movie 6. Micromotor self-propulsion at 200 mM urea for different concentrations of NaOH (AVI).

Movie 7. Micromotor self-propulsion at 200 mM urea for different concentrations of HEPES (AVI).

Movie 8. Micromotor self-propulsion at 200 mM urea for different concentrations of NaCl (AVI).

Movie 9. Micromotor Brownian motion at 0 mM urea for different concentrations of PBS, NaOH, NaCl and HEPES (AVI).

1. Dunderdale, G., Ebbens, S., Fairclough, P. & Howse, J. Importance of particle tracking and calculating the mean-squared displacement in distinguishing nanopropulsion from other processes. *Langmuir* **28**, 10997–11006 (2012).

2. Patton, C. J. & Crouch, S. R. Spectrophotometric and kinetics investigation of the Berthelot reaction for the determination of ammonia. *Anal. Chem.* **49**, 464–469 (1977).
